# *Lamprops donghaensis* sp. n. (Crustacea, Cumacea, Lampropidae), a new species from Korean waters

**DOI:** 10.3897/zookeys.517.10097

**Published:** 2015-08-12

**Authors:** Sung-Hyun Kim, Young-Hyo Kim

**Affiliations:** 1 Department of Life Sciences, Dankook University, Cheonan, Korea 330-714

**Keywords:** Cumacea, Lampropidae, *Lamprops*, new species, Korea, key, taxonomy

## Abstract

A new species of Cumacea belonging to the genus *Lamprops* Sars was collected from the East Sea of Korea. This new species resembles *Lamprops comatus* Zimmer, *Lamprops carinatus* Hart, *Lamprops flavus* Harada, *Lamprops pumilio* Zimmer, *Lamprops tomalesi* Gladfelter, and *Lamprops obfuscatus* (Gladfelter) in lacking lateral oblique ridges on the carapace and lateral setae on the telson. The new species, however, is distinguished from its congeners by having a dorsal concave groove and a lateral rounded depressed area on pereonite 2. The new species is fully illustrated and compared with related species. A key to the world *Lamprops* species lacking lateral ridges on the carapace is also provided.

## Introduction

The genus *Lamprops* Sars, 1863, belonging to the family Lampropidae, commonly inhabits cool water, is bipolar in distribution and is also a shallow water marine benthos (Day 1978; Tsareva and Kepel 2001). This genus is morphologically characterized by having a distinct antennal notch, telson with 3-5 apical setae and male lacking pleopods (Given 1964; Gamô 1967; Kim et al. 2015). To date, 22 species have been reported worldwide (Tzareva and Vassilenko 2006; Roccatagliata and Mühlendhardt-Siegel 2012; WoRMS 2015). For the study on the Korean lampropid species, lampropid specimens were collected from the East Sea of Korea. Recently, two *Lamprops* species, *Lamprops carinatus* Hart, 1930 and *Lamprops pseudosarsi* Tsareva & Vassilenko, 1993 were reported for the first time in Korean waters (Kim et al. 2015) and here we describe and illustrate a new species of the genus. Therefore, a total of four species of the lampropid species including *Hemilamprops californicus* (Zimmer, 1936) are reported from Korea.

## Material and methods

The specimens were collected using a light-trap (Holmes and O’Connor 1988; Kim 1992) from shallow water at Geojin Port, Goseong-gun, Gangwon-do, Korea. The specimens were fixed in 70–80% ethanol and dissected in glycerol on cobb’s aluminum hole slides. Drawings and measurements were performed with the aid of a drawing tube. Measurements for the body length were made from the anterior tip of the carapace to the last abdominal segment and for each appendage were made along the mid–line of the articles, exclusive of the inflated outer angle. Type specimens were deposited at the National Institute of Biological Resources (NIBR), Incheon, Korea and at the Department of Biological Science, Dankook University (DKU), Cheonan, Korea. The terminology for the setae follows that used by Watling (1989) and Gerken (2010, 2013).

## Taxonomy

### Genus Lamprops

Taxon classificationAnimaliaCumaceaLampropidae

Sars, 1863

#### Type species.

*Lamprops fasciatus* Sars, 1863

#### Species composition.

*Lamprops affinis* Lomakina, 1958; *Lamprops augustinensis* Gerken, 2005; *Lamprops beringi* Calman, 1912; *Lamprops carinatus* Hart, 1930; *Lamprops comatus* Zimmer, 1907; *Lamprops fasciatus* G.O. Sars, 1863; *Lamprops flavus* Harada, 1959; *Lamprops fuscatus* Sars, 1865; *Lamprops hexaspinula* Liu & Liu, 1990; *Lamprops kensleyi* Haye & Gerken, 2005; *Lamprops korroensis* Derzhavin, 1923; *Lamprops lomakinae* Tsareva & Vassilenko, 1993; *Lamprops multifasciatus* Zimmer, 1937; *Lamprops obfuscatus* (Gladfelter, 1975); *Lamprops pseudosarsi* Tsareva & Vassilenko, 1993; *Lamprops pumilio* Zimmer, 1937; *Lamprops quadriplicatus* S.I. Smith, 1879; *Lamprops sarsi* Derzhavin, 1926; *Lamprops serratus* Hart, 1930; *Lamprops tenuis* Tzareva & Vassilenko, 2006; *Lamprops tomalesi* Gladfelter, 1975; and *Lamprops triserratus* (Gladfelter, 1975).

### Lamprops donghaensis sp. n.

Taxon classificationAnimaliaCumaceaLampropidae

http://zoobank.org/06DB8C52-2FE2-4509-BE5D-59560D13C29E

Korean name: Dong-Hae-sap-kko-ri-ol-chaeng-i-sae-u, new

**Figure 1.**
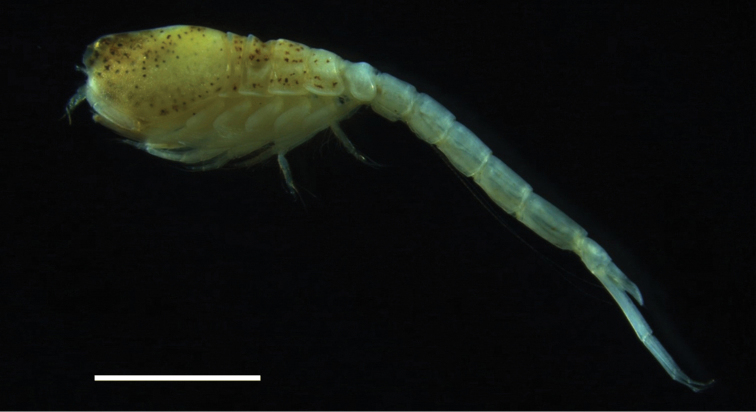
*Lamprops donghaensis* sp. n., paratype, male, 7.6 mm, Geojin Port, Geojin-eup, Goseong-gun, Gangwon-do, Korea. Scale bar: 2.0 mm.

**Figure 2.**
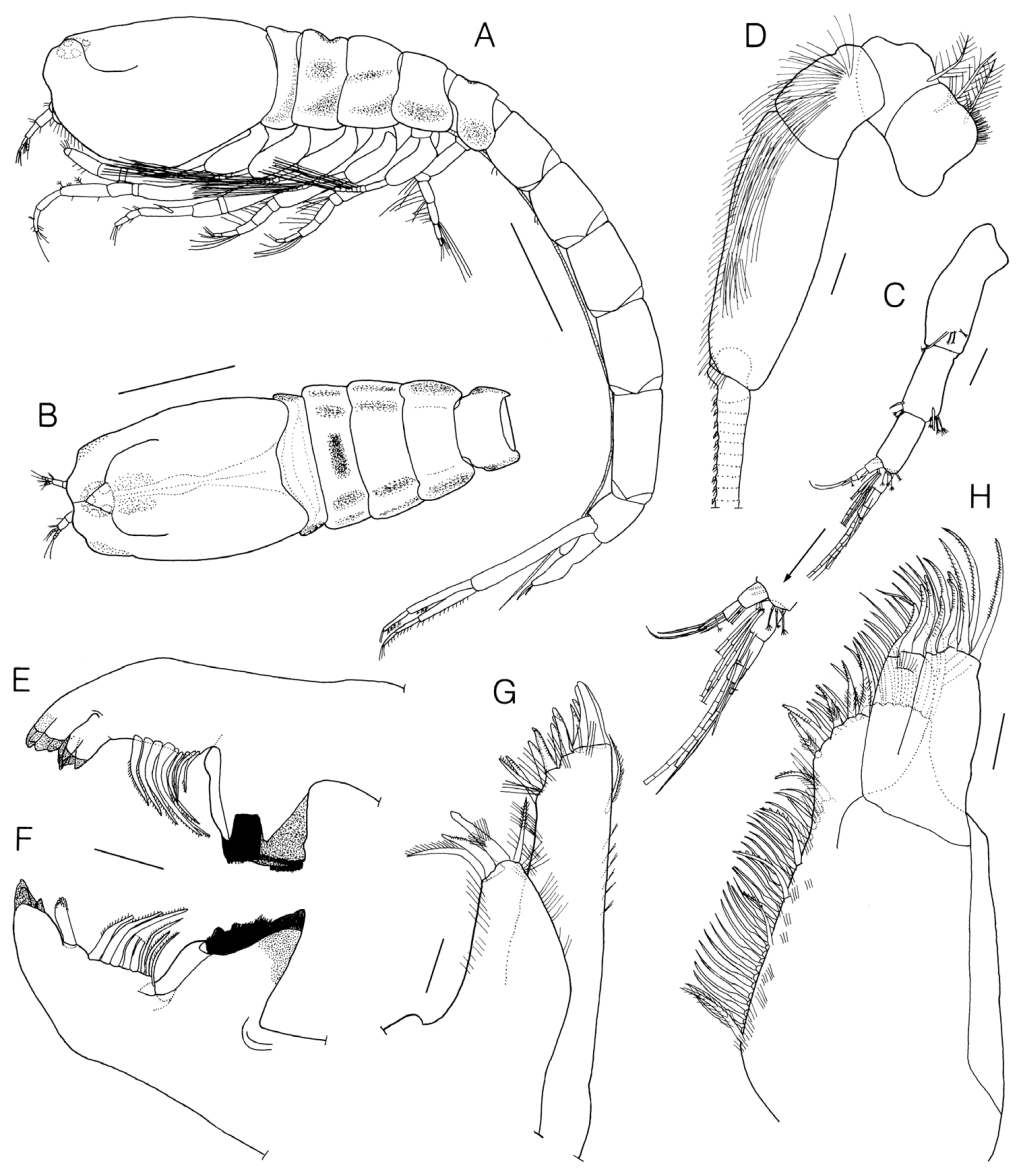
*Lamprops donghaensis* sp. n., holotype, male, **A** habitus, lateral **B** cephalothorax, dorsal (from paratype, 7.6 mm) **C** antenna 1 **D** antenna 2 **E** left mandible **F** right mandible **G** maxilla 1 **H** maxilla 2. Scale bars: 1.0 mm (**A, B**), 0.1 mm (**C–F**), 0.05 mm (**G, H**).

**Figure 3.**
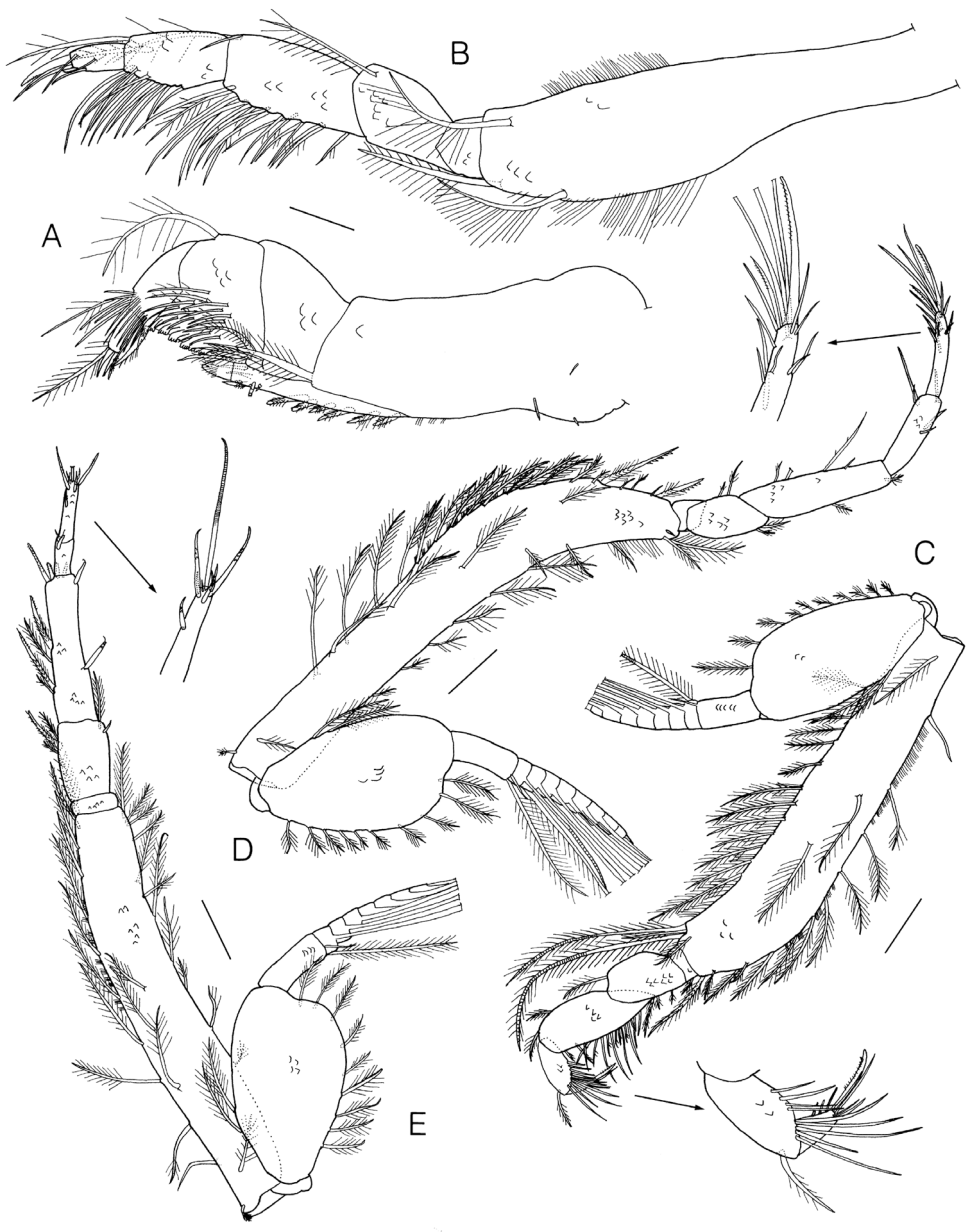
*Lamprops donghaensis* sp. n., holotype, male, **A** maxilliped 1 **B** maxilliped 2 **C** maxilliped 3 **D** pereopod 1 **E** pereopod 2. Scale bars: 0.2 mm (**C–E**), 0.1 mm (**A, B**).

**Figure 4.**
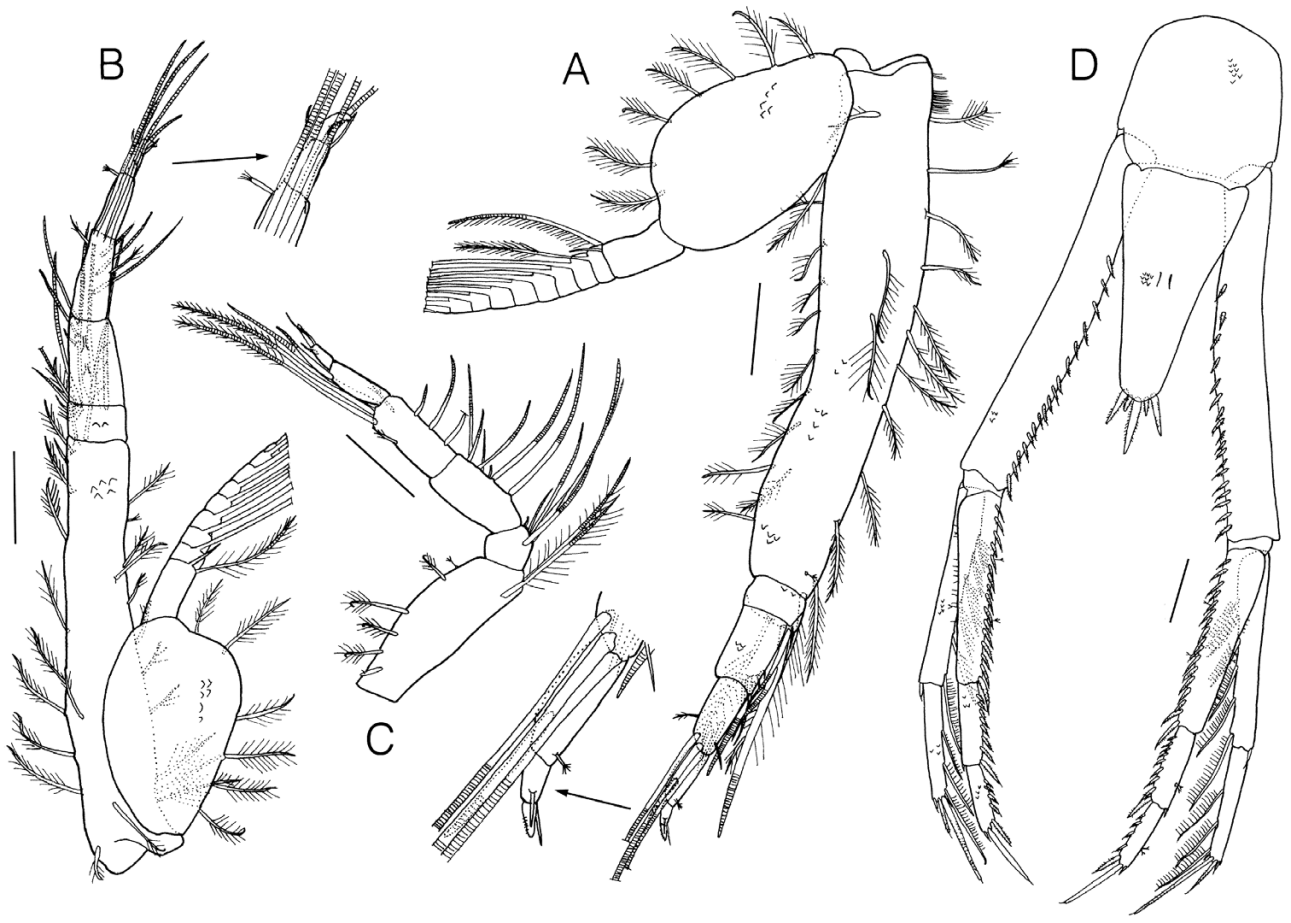
*Lamprops donghaensis* sp. n., holotype, male. **A** pereopod 3 **B** pereopod 4 **C** pereopod 5 **D** Telson and uropod. Scale bars: 0.2 mm.

**Figure 5.**
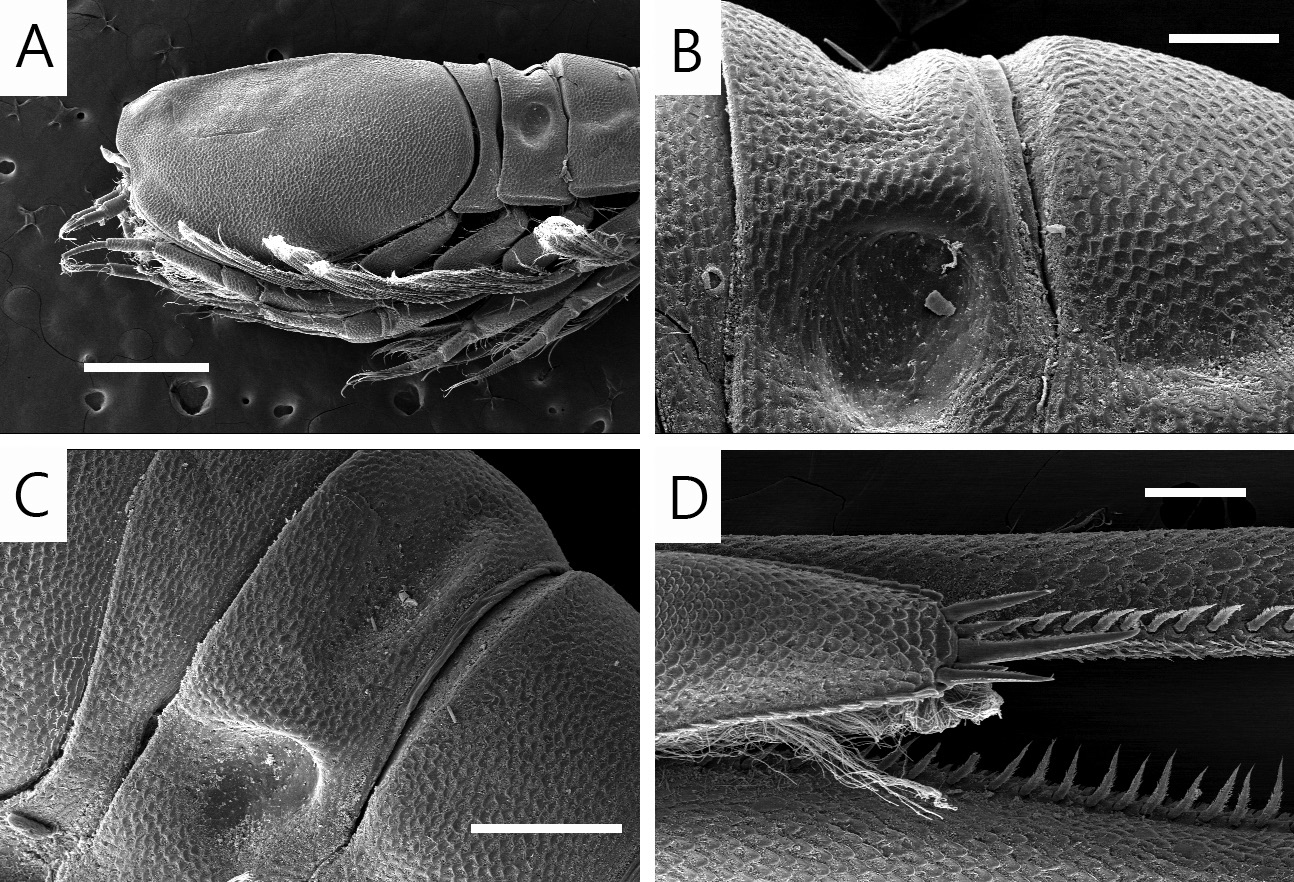
*Lamprops donghaensis* sp. n., paratype, **A** carapace, lateral view **B** pereonite 2, lateral view **C** pereonite 2, dorsal view **D** telson and uropod. Scale bars: 0.5 mm (**A**), 0.25 mm (**C**), 0.125 mm (**B**), 0.1 mm (**D**).

Figures 1 , 2 , 3 , 4 , 5

#### Type material.

Holotype: adult male, 7.9 mm, NIBRIV0000317121, Geojin Port, Geojin-eup, Goseong-gun, Gangwon-do, Korea, 38°26'44"N 128°27'40"E, S.S. Hong and S.H. Kim, 11 April 2013. Paratypes: 320 males, 7.6–8.9 mm, DKUCUM 201501, 11 April 2013, same station data as holotype.

#### Additional material examined.

5 males, 7.9–8.4 mm, 15 February 2012, same station data as holotype; 1 male, 8.0 mm, Gangneung Port, Gyeonso-dong, Gangneung-si, Gangwon-do, Korea, 37°46'15.9"N, 128°57'05.2"E, S.S. Hong and S.H. Kim, 30 March 2012; 1 male, 8.3 mm, Cheongchoho, Cheongcho-dong, Sokcho-si, Gangwon-do, Korea, 38°12'01.7"N, 128°35'37.2"E, S.S. Hong and S.H. Kim, 12 April 2013; 9 males, 7.7–8.7 mm, 15 February 2014, same station data as holotype; 1 male, 8.6 mm, Oeongchi Port, Daepo-dong, Sokcho-si, Gangwon-do, Korea, 37°46'15.9"N, 128°57'05.2"E, S.S. Hong and S.H. Kim, 30 March 2012.

#### Description.

**Holotype, adult male**, NIBRIV0000317121.

Body (Fig. 2A) 7.9 mm long, surface with a scale-like sculpturing. Carapace (Fig. 2A, 2B) smooth, without oblique ridges, subovate in lateral view, subrectangular in dorsal view, 1.35 × wide, 0.23 × body, subequal to pereonites 1–5, dorsal carina reaching 0.94 × distal end of carapace. Pereonite 2 (Fig. 2A) with dorsal transverse groove, concave dorsomesially in lateral view, lateral portion with concave rounded area.

Antenna 1 (Fig. 2C) peduncle triarticulate; proximal article subequal to remaining articles combined, with 1 simple and 3 complex pedunculate setae subdistally; article 2 0.55 × proximal article, with 5 simple and 4 complex pedunculate setae distally; distal article 0.78 × article 2, with 2 simple and 3 complex pedunculate setae; main flagellum 4-articulated, with 5 aesthetascs and 6 simple setae; accessory flagellum short, 3-articulated, with 8 simple and 1 complex pedunculate setae.

Antenna 2 (Fig. 2D) elongate, slightly extending beyond end of telson; peduncle 5-articulated, article 2 stubby, subequal to article 3, with 2 plumose setae and short setules; articles 4–5 with numerous simple setae; each article of flagellum with 1 or 2 small simple setae.

Left mandible (Fig. 2E) boat-shaped, incisor with 4 cusps, with row of 9 lifting setae and lacinia mobilis.

Right mandible (Fig. 2F) similar to left one except incisor with 3 cusps and lacking lacinia mobilis.

Maxilla 1 (Fig. 2G) outer endite with row of 2 stout simple, 10 stout microserrate, and 1 stout serrate setae terminally, tufts of setules subterminally, 1 pappose seta and 6 setules on lateral margin; inner endite approximately half length of outer, with 1 pappose, 1 stout pappose, 1 stout microserrate, and 1 plumose setae terminally.

Maxilla 2 (Fig. 2H) broad endite with 8 plumose, 13 simple, 4 papposerrate, and 1 microserrate setae terminally, medial face with a row of 30 simple, 1 papposerrate, 3 serrate, 1 pappose, and hair-like setae; each outer and inner narrow endite with 7 or 3 stout microserrate setae terminally.

Maxilliped 1 (Fig. 3A) basis subrectangular, subequal to the following articles combined, medial lobe with 2 hook, 6 pappose, and hair-like setae medially, 1 stout knoblike, 2 simple, and 1 pappose setae distally; ischium absent; merus with 3 pappose setae medially; carpus subequal to merus, with plumose, simple, and comb-like setae medially, 1 plumose seta laterally; propodus with 4 plumose, 1 pappose, 1 papposerrate, and numerous simple setae distally; dactylus with 2 simple setae terminally.

Maxilliped 2 (Fig. 3B) basis elongate, longer than remaining articles combined, with 3 plumose and hair-like setae; ischium short, unarmed; merus 0.80 × carpus, with 1 plumose seta distally; capus with 11 plumose and 7 simple setae medially, 1 plumose and 1 simple setae laterodistally; propodus 0.80 × carpus, with 13 simple setae medially, 2 plumose setae distally; dactylus 0.51 × propodus, with 1 stout microserrate and 5 simple setae.

Maxilliped 3 (Fig. 3C) basis much longer than remaining articles combined, with 1 simple, 11 plumose setae, and tufts of setules posteriorly, 12 plumose and hair-like setae anteriorly, 2 plumo-annulate and 1 plumose setae anterodistally; ischium very short, with 1 small plumose seta posteriorly; merus 0.69 × carpus, with 1 pappose and 2 plumose setae posteriorly, 1 plumose seta anterodistally; carpus with 9 plumose and 7 simple setae posteriorly, 2 plumose setae anterodistally; propodus 0.47 × carpus, with 10 simple setae posteriorly, 1 plumose seta anterodistally; dactylus with 1 stout microserrate seta terminally, and 6 simple setae subterminally; exopod shorter than basis, flagellum with 1 simple and numerous plumo-annulate setae.

Pereopod 1 (Fig. 3D) basis somewhat curved, 1.29 × remaining articles combined, with 17 plumose, 2 papposerrate, 1 small simple setae, and tufts of setules posteriorly, 7 plumose and some hair-like setae anteriorly, 2 plumose and 1 small setae anterodistally; merus 0.45 × carpus, with 2 plumose setae posteriorly and anterodistally; propodus 0.64 × carpus, with 5 simple setae; dactylus 0.93 × propodus, with 2 microserrate and 12 simple setae, terminal seta elongate, slightly shorter than dactylus; exopod shorter than basis, flagellum with 1 simple and numerous plumo-annulate setae.

Pereopod 2 (Fig. 3E) basis slightly curved, 1.25 × remaining articles combined, with 1 simple, 8 plumose, 1 pappose setae, and tufts of setules posteriorly, row of 11 plumose setae anteriorly; carpus subrectangular, 1.96 × merus, with 2 plumose and 2 papposerrate setae posteriorly, 1 microserrate seta with single subapical setule anteriorly, 4 microserrate and 1 simple setae terminally; propodus short, 0.26 × carpus, with 1 simple seta with single subterminal setule; dactylus 1.72 × propodus, with 4 microserrate and 3 simple setae; exopod shorter than basis, flagellum with 1 simple and numerous plumo-annulate setae.

Pereopod 3 (Fig. 4A) basis longer than remaining articles combined, with 10 plumose setae posteriorly, 3 plumose and 1 complex pedunculate setae on lateral surface, 8 plumose setae anteriorly, and 2 plumose setae on medial surface; ischium short, 0.51 × merus, with 4 annulate, 1 simple, and 1 plumose setae; merus 0.95 × carpus, with 4 annulate and 1 small simple setae posterodistally; propodus 0.77 × carpus, with 1 annulate and 1 complex pedunculate setae on lateral surface; dactylus 0.33 × propodus, with 1 simple seta on lateral surface, 1 stout microserrate and 1 simple setae terminally; exopod shorter than basis, flagellum with 1 simple and numerous plumo-annulate setae.

Pereopod 4 (Fig. 4B) basis longer than remaining articles combined, with 8 plumose and 1 complex pedunculate setae posteriorly, 3 plumose setae on lateral surface, 9 plumose and 1 complex pedunculate setae anteriorly, 2 plumose setae mediodistally; merus subequal to carpus, with 4 annulate and 1 simple setae on medial surface; carpus 1.34 × propodus; propodus longer than dactylus, with 1 annulate seta on lateral surface, 1 complex pedunculate seta posterodistally; exopod subequal to basis, flagellum with 1 simple and numerous plumo-annulate setae.

Pereopod 5 (Fig. 4C) basis subrectangular, 0.60 × remaining articles combined, with 5 plumose, 1 complex pedunculate, and 2 long plumo-annulate setae; ischium 0.40 × merus, with 5 annulate and 1 small simple setae anterodistally; merus subequal to carpus, with 5 annulate and 2 simple setae anteriorly; carpus 1.47 × propodus, with 3 annulate setae anteriorly, 1 long annulate and 2 long plumo-annulate setae posterodistally; propodus with 1 annulate seta posterodistally; dactylus 0.42 × propodus; exopod absent.

Telson (Fig. 4D) equilaterally triangular, width 0.53 × length, 1.42 × pleonite 6, without lateral setae, with 2 simple setae dorsomesially, 5 stout microserrate distal setae of which middle one is longest, a pair of neighboring setae short, 0.31 × middle one, the distolateral setae 0.74 × middle one.

Uropodal peduncle (Fig. 4D) 1.66 × telson, with a row of 17–18 small stout microserrate setae medially; endopod triarticulate, 0.87 × peduncle; proximal article 2.27 × article 2, with 16–17 small stout microserrate and 2 complex pedunculate setae medially; article 2 1.22 × distal article, with 8–9 small stout microserrate setae medially; distal article with 4 small stout microserrate setae medially, 1 stout microserrate and 2 unequal simple setae terminally; exopod biarticulate, slightly shorter than the endopod, proximal article 1.69 × article 2, with 6 plumose setae medially and 1 small simple seta on lateral distal corner; article 2 with 3 plumose setae medially, 2 small simple setae and 2 microserrate setae terminally.

Female. Unknown.

#### Remarks.

This new species resembles *Lamprops comatus* Zimmer, 1907, *Lamprops carinatus* (Hart, 1930), *Lamprops flavus* (Harada, 1959), *Lamprops pumilio* (Zimmer, 1937), *Lamprops tomalesi* Gladfelter, 1975, and *Lamprops obfuscatus* (Gladfelter, 1975) in lacking an oblique ridges on the carapace and lateral setae on the telson. *Lamprops donghaensis* sp. n., however, is distinguished from its congeners by the dorsal concave groove and lateral concave depressed area on pereonite 2. The characteristics are listed in Table 1 as well as in the key. The new species is more similar to *Lamprops carinatus* in having a similar medium-sized body, a similar terminal setae type of telson, and similar length ratio for the uropodal exopod and endopod (see Hart 1930, and Kim et al. 2015). However, the new species is distinguished from *Lamprops carinatus* by the combination of the following features (*Lamprops carinatus* condition in parentheses): 1) pereonite 2 concave dorsally, with dorsal transverse groove and lateral rounded depressed area (flat dorsally, without dorsal groove and lateral depressed area); 2) maxilliped 3, basis with a row of plumose setae anteriorly (without plumose setae anteriorly); 3) telson 1.48 × pleonite 6 (1.31 × pleonite 6); 4) uropodal peduncle with 17–18 small stout microserrate setae (with 11 setae); 5) uropodal endopod, distal article with 4 microserrate setae medially (without microserrate seta).

**Table 1.** Comparison of morphological characteristics among *Lamprops donghaensis* sp. n. and related species.

Characteristics and distribution	Species					
	*Lamprops donghaensis* sp. n. (male)	*Lamprops carinatus* (male)	*Lamprops flavus* (male)	*Lamprops pumilio* (male)	*Lamprops tomalesi* (female)	*Lamprops obfuscatus* (female)
Body length (mm)	7.6–8.9	6.0–7.9	2.6 (without telson)	3.5	4.0	4.0
Dorsomedian carina	0.94 × carapace	0.88 × carapace	?	?	?	?
Pereonite 2, dorsal side	concave	flat	flat	flat	flat	flat
Pereonite 2, lateral side	with rounded area	without rounded area	without rounded area	without rounded area	without rounded area	without rounded area
Maxilliped 3, anterior margin of basis	with plumose setae	without plumose setae	?	?	with plumose setae	?
Antenna 2, length	more than telson	reaching base of the telson	reaching middle of the pleonite 5	reaching end of the thorax	vestigial	vestigial
Pereopod 1, basis	1.29 × remaining articles combined	1.30 × remaining articles combined	?	?	0.87 × remaining articles combined	1.29 × remaining articles combined
Uropodal peduncle, number of inner setae	17–18	11	12	8–10	8–10	4
Uropod, exopod length	0.91 × endopod	0.98 × endopod	0.95 × endopod	?	0.92 × endopod	0.79 × endopod
Uropodal endopod, distal article setae	2–4 medial stout setae	without medial setae	?	?	without medial setae	without medial setae
Distribution	Korea (present study)	Korea (Kim et al. 2015), Alask, Vancouver (Lomakina 1958), Gabriola Island (Hart 1930)	Shimoda Bay (Harada 1959)	South Kuril Islands, Okhotsk Sea (Lomakina 1958)	California (Gladfelter 1975)	California (Gladfelter 1975)

#### Etymology.

The specific epithet *donghaensis* originates from the Korean word “Dong-Hae”, meaning the East Sea, named after the eastern Korean coast in which the species was discovered.

#### Habitat.

The new species was collected together with *Lamprops carinatus* and *Lamprops pseudosarsi* at the same location, in Geojin Port, Goseong-gun, Korea, which is a sandy substrate.

#### Distribution.

Geojin Port, Geojin-eup, Goseong-gun, Gangwon-do, Korea.

### Key to the species of genus *Lamprops* (without oblique ridge of carapace)

**Table:** 

1	Telson with lateral setae	**2**
–	Telson without lateral setae	**5**
2	Telson with 2 pairs of lateral setae	***Lamprops fuscatus* Sars, 1865**
–	Telson with more than 2 pairs of lateral setae	**3**
3	Telson with 5 or 6 lateral setae	***Lamprops serratus* Hart, 1930**
–	Telson with 4 pairs of lateral setae	**4**
4	Telson with 3 apical setae	***Lamprops kensleyi* Haye & Gerken, 2005**
–	Telson with 5 apical setae	***Lamprops affinis* Lomakina, 1958**
5	Telson without lateral serration	**6**
–	Telson with lateral serration	***Lamprops comatus* Zimmer, 1907**
6	Body small, < 4.0 mm	**7**
–	Body medium, ≥ 4.0 mm	**8**
7	Carapace, anteroventral corner subquadrate	***Lamprops flavus* Harada, 1959**
–	Carapace, anteroventral corner rounded	***Lamprops pumilio* Zimmer, 1937**
8	Telson, lateral apical setae longest	***Lamprops korroensis* Derzhavin, 1923**
–	Telson, middle apical seta longest	**9**
9	Telson, apicolateral setae shortest	***Lamprops tomalesi* Gladfelter, 1975**
–	Telson, apicolateral setae not shortest	**10**
10	Pereonite 2 concave dorsally, with dorsal groove and lateral rounded depressed area	***Lamprops donghaensis* sp. n.**
–	Pereonite 2 flat dorsally, without dorsal groove and lateral rounded depressed area	**11**
11	Uropodal peduncle with 6–11 inner setae	***Lamprops carinatus* Hart, 1930**
–	Uropodal peduncle with 4 inner setae	***Lamprops obfuscatus* (Gladfelter, 1975)**

## Supplementary Material

XML Treatment for Lamprops

XML Treatment for Lamprops donghaensis
